# Cadherin-11 in poor prognosis malignancies and rheumatoid arthritis: common target, common therapies

**DOI:** 10.18632/oncotarget.1538

**Published:** 2013-11-15

**Authors:** Shahin Assefnia, Sivanesan Dakshanamurthy, Jaime M. Guidry Auvil, Constanze Hampel, Panos Z. Anastasiadis, Bhaskar Kallakury, Aykut Uren, David W Foley, Milton L. Brown, Lawrence Shapiro, Michael Brenner, David Haigh, Stephen W. Byers

**Affiliations:** ^1^ The Georgetown-Lombardi Comprehensive Cancer Center, Georgetown University Medical Center, Washington, DC, USA; ^2^ Department of Cancer Cell Biology, Mayo Clinic, Jacksonville, FL, USA; ^3^ Department of Medicine, Division of Rheumatology, Immunology, and Allergy, Brigham and Women's Hospital, Boston, MA. USA; ^4^ Center for Cancer Research and Cell Biology, School of Medicine, Dentistry and Biomedical sciences, Queen's University Belfast, Northern Ireland, UK; ^5^ Center for Drug Discovery, Georgetown University Medical Center,Washington, DC, USA; ^6^ Department of Biochemistry and Molecular Biophysics, Columbia University, New York, NY, USA

**Keywords:** cadherin-11, breast cancer, glioblastoma, small molecule inhibitor, rheumatoid arthritis, celecoxib

## Abstract

Cadherin-11 (CDH11), associated with epithelial to mesenchymal transformation in development, poor prognosis malignancies and cancer stem cells, is also a major therapeutic target in rheumatoid arthritis (RA). CDH11 expressing basal-like breast carcinomas and other CDH11 expressing malignancies exhibit poor prognosis. We show that CDH11 is increased early in breast cancer and ductal carcinoma in-situ. CDH11 knockdown and antibodies effective in RA slowed the growth of basal-like breast tumors and decreased proliferation and colony formation of breast, glioblastoma and prostate cancer cells. The repurposed arthritis drug celecoxib, which binds to CDH11, and other small molecules designed to bind CDH11 without inhibiting COX-2 preferentially affect the growth of CDH11 positive cancer cells in vitro and in animals. These data suggest that CDH11 is important for malignant progression, and is a therapeutic target in arthritis and cancer with the potential for rapid clinical translation

## INTRODUCTION

Poor prognosis epithelial-derived cancers often exhibit morphologic and molecular changes characteristic of an epithelial to mesenchymal transition (EMT) and EMT markers are predominantly found in tumors with a basal-like phenotype [1;2]. Breast cancer cell lines can be divided into subtypes that parallel clinical response [3]. Basal B lineage cells are poorly differentiated, exhibit mesenchymal morphology, and are frequently highly aggressive and invasive. Increased expression of the mesenchymal cadherins N-cadherin and/or cadherin-11 (CDH11) and decreased E-cadherin, have been associated with both EMT and tumor progression [1;4]. CDH11 is expressed only in poorly differentiated, highly-invasive cells [5]. All CDH11 positive cell lines are in the basal B subset of poor prognosis breast cancer cells [3]. Importantly, CDH11 is a therapeutic target in rheumatoid arthritis (RA), an inflammatory disease with properties often compared with cancer. Systemic administration of CDH11 antibodies reverses the proliferation and migration of synoviocytes to the sites of joint inflammation and attenuates symptoms of RA [6]. As CDH11 antibody based therapeutics are in clinical trials for RA and we showed recently that the arthritis drug celecoxib has the structural potential to bind CDH11, there is a strong possibility that, if CDH11 can be shown to drive malignant progression rather than simply be associated with it, therapeutic options may be rapidly developed [7]. For example, CDH11 expression promotes the formation of skeletal metastases in models of prostate cancer and can regulate glioma survival and migration [8;9]. To more formally test the association of CDH11 with poor prognosis malignancies we first carried out a meta-analysis of all published datasets to show that in addition to being elevated in early stages of breast cancer such as ductal carcinoma in-situ (DCIS), CDH11 is highly expressed in gastrointestinal, brain and central nervous system tumors. We go on to show that CDH11 is necessary for MDA-MB-231 cell tumor growth and that it regulates proliferation, colony formation, migration and invasion of several CDH11 positive tumor cells. Finally, CDH11 is a type II cadherin, bearing two tryptophan residues with distinctively large hydrophobic pockets in its extracellular domain 1 (EC1) binding domain [10]. As type I cadherins such as E and N-cadherins, only have one tryptophan residue in their binding pockets, CDH11 offers a somewhat unique domain for targeting. We showed previously that the arthritis drug celecoxib had the structural potential to bind this pocket and now show that celecoxib, a celecoxib analogue with no COX-2 inhibitory activity, as well as several novel small molecules can selectively inhibit the growth of CDH11 expressing breast cancer cells [7].

## RESULTS

### CDH11 is increased in early stages of human breast cancer and in other malignancies

**Figure 1:**
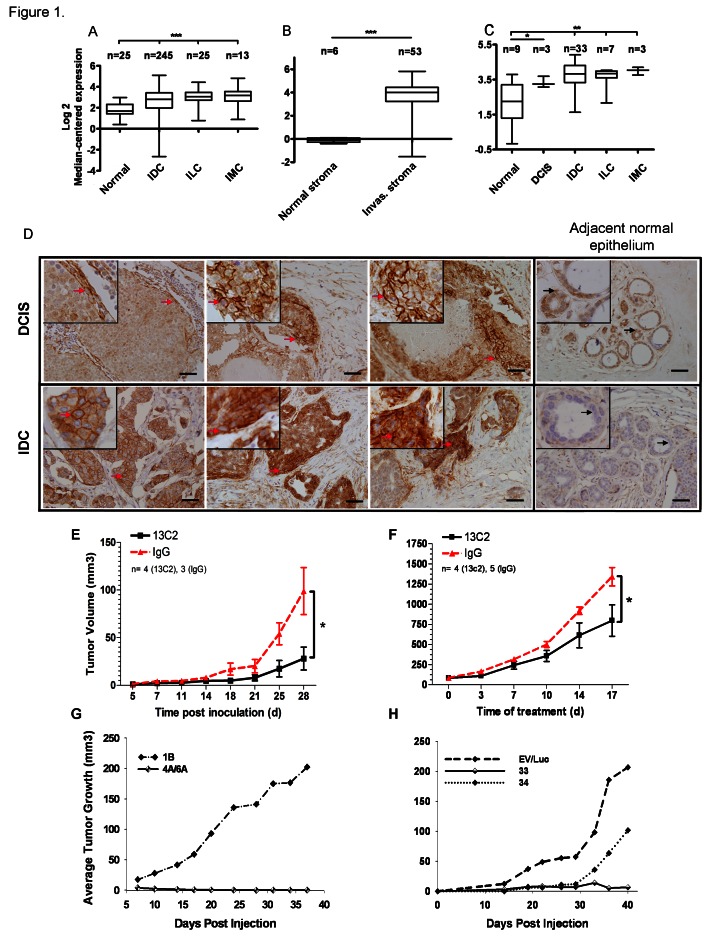
CDH11 mRNA and protein are expressed in ductal carcinoma in situ and invasive breast carcinoma and regulates growth of MDA-MB-231 breast cancer transplanted cells (A) CDH11 transcript is significantly increased in invasive carcinoma compared to normal breast (http://cancergenome.nih.gov), (B) and in invasive cancer stroma compared to normal stroma [11]. (C) Significant CDH11 up-regulation is also seen as an early event in ductal carcinoma in-situ [12]. The fold changes (FC) in the expression level of CDH11 and statistical analysis p values (Welch's t-test) were as follows: (A) Invasive ductal carcinoma, invasive lobular carcinoma and invasive mixed carcinoma vs. normal breast: p<0.0001 for all, FC: 1.75, 2.27, 2.44 respectively, (B) Invasive breast carcinoma stroma vs. normal breast stroma: p<0.0001, FC: 14.239. (C) Ductal carcinoma in-situ vs. normal breast: p=0.0194, FC: 2.160, invasive ductal carcinoma vs. normal breast: p=0.0034, FC: 2.896, invasive lobular carcinoma vs. normal breast: p=0.0077, FC: 2.603, invasive mixed carcinoma vs. normal breast: p=0.0015, FC: 3.413. Data presented as bars and whiskers (D) CDH11 immunohistochemical staining of human DCIS (left to right: comedo-type, cribriform and another comedo-type) and invasive ductal carcinoma as well as adjacent normal epithelium. (E) Growth inhibition of CDH11 positive MDA-MB-231 xenografts (>70%) upon treatment with 13C2 or control IgG with 0.5 mg initial I.P. injection followed by 0.1 mg subsequent injections (x3/week) for a month. (p=0.0365 compared to control (IgG), 28 d, two-tailed student's t-test). (F) Growth inhibition of established MDA-MB-231 tumors (>40%) upon I.P. treatment with 13C2 antibody or control (IgG) at 20 mg per kg body weight (x2/week) beginning when subcutaneous tumors were palpable. (p=0.0394 for 13C2 compared to control (IgG), 17 d post treatment start, two-tailed student's t-test). (G,H): Inhibitory effect of CDH11 knockdown on tumor growth in mice. Athymic nude mice were s. c. injected with 1-2×10^6^ MDA-MB-231 cells stably expressing (G) CDH11 siRNA, or (H) shRNA or controls into 1 of 4 mammary fat pads, such that all cell lines were represented in a minimum 10 total injections each (minimum of 2x per specific locus). For ILC images, please see Supplementary Fig. S2. The red arrows indicate CDH11 membranous staining. Black arrows point to a cell within the region that is magnified in the small insets. Scale bar: 45 μM. Small insets are 2.5x magnification of large images. Data are presented as means ± SEM. IDC, ILC and IMC: Invasive ductal, lobular and mixed carcinomas respectively, Invas: invasive.

We performed a meta-analysis of all publicly available human cancer microarray datasets, including The Cancer Genome Atlas (TCGA) (http://cancergenome.nih.gov) containing both normal and tumor information using Oncomine™. CDH11 is increased in invasive breast cancers (Figure 1A-C, Supplementary Fig. S1B-E) [11-16] and in DCIS when compared to normal tissue (Figure 1C, Supplementary Fig. S1E)[12;16]. CDH11 is markedly elevated in the stroma of invasive breast cancers compared to normal stroma (Figure 1B, supplementary Fig. S1D) [11;15]. These data suggest that increased CDH11 is an early event in breast cancer progression. In addition to breast cancer and DCIS, CDH11 was increased in most data sets from brain and central nervous system (CNS), and gastrointestinal malignancies (Supplementary Fig. S1A).

For immunohistochemistry we used an antibody that exhibits no cross-reactivity with other cadherin family members [17] (please see materials and methods). Some DCIS lesions were completely negative for CDH11 expression, in others CDH11 was expressed on the periphery of the lesion while in others CDH11 positive cells extended into the lumen. Comedocarcinoma was the most common subtype in the positive DCIS foci. CDH11 was expressed at high levels in most invasive ductal carcinoma cells (IDCs) (Figure 1D). However, in almost all invasive lobular carcinomas (ILCs) CDH11 expression was limited to the stroma (Supplementary Fig. S2.), with the exception of, pleomorphic ILC in which CDH11 staining was present throughout occasional cells of the ILC itself (Supplementary Fig. S2A). Pleomorphic ILCs are more invasive in nature with poor prognosis compared to other ILCs [18].

### Tumor-cell CDH11 is required for subcutaneous growth *in vivo*

MDA-MB-231 CDH11-expressing breast cancer cells were inoculated into nude mice and treated Matrigel™ with a function-blocking monoclonal CDH11-specific antibody that does not exhibit significant side-effects in inflammatory RA, or control IgG [6]. Anti-CDH11 antibody therapy significantly inhibited the growth of newly injected xenografts (Figure 1E) and of established tumors compared to control mice (Figure 1F).

Although CDH11 is elevated in cancer stromal tissue (Figure 1B and Supplementary Fig. S1D) [11;15], stromal tissue likely contains tumor cells in the process of invasion as well as host stromal cells and it is not clear which population contributes to the observed increases in mRNA. To address this, we used siRNA to knock down CDH11 in MDA-MB-231 cells. Cells containing either one of two different siRNA CDH11 target sequences completely failed to form tumors in nude mice (n=20) (Figure 1G). Stable cell lines containing shRNA target sequences displayed a significant delay to onset of tumor growth compared to empty vector or scrambled controls. Tumor growth was observed in one lentivirally-infected line approximately one month after inoculation (Figure 1H) but these cells were found to be re-expressing CDH11 *in vitro* as measured by Western blot (data not shown). These data, along with the functional assays *in vitro*, strongly suggest that tumor cell CDH11 is necessary for MDA-MB-231 tumor growth.

### CDH11 depletion alters cell number, colony formation, migration and Matrigel™ outgrowth of invasive cancer cells

**Figure 2:**
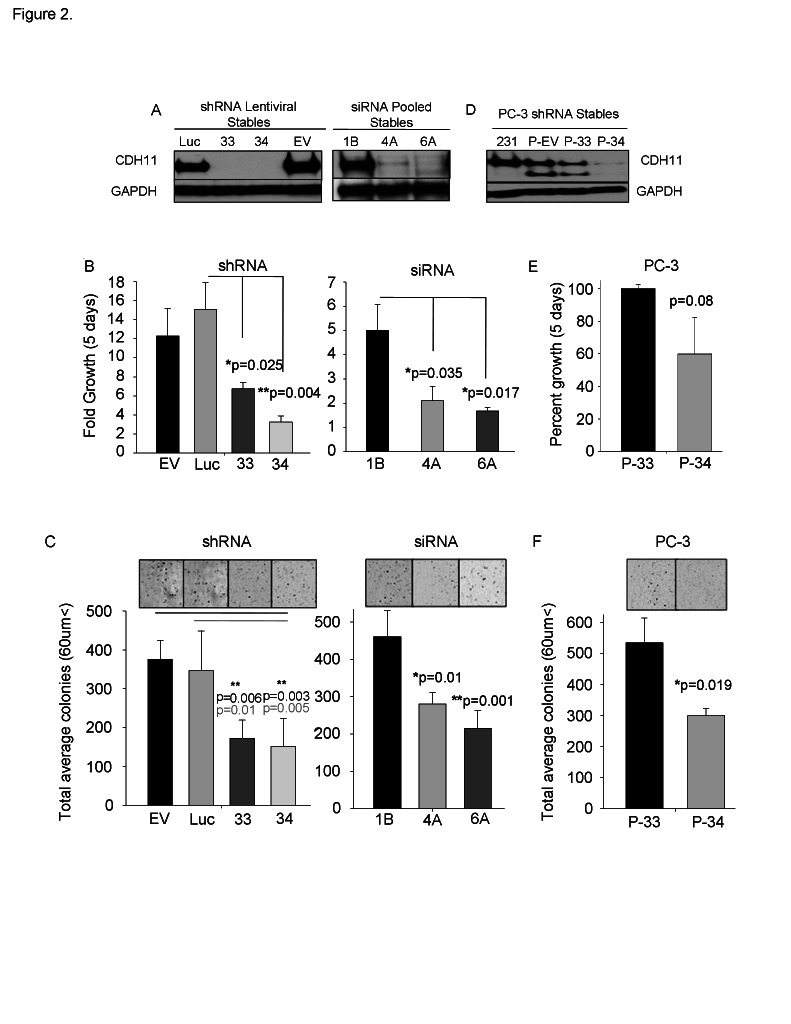
CDH11 regulates colony growth and proliferation of MDA-MB-231 breast cancer cells and PC-3 prostate cancer cells (A) Western blot analysis of CDH11 in MDA-MB-231 cells stably expressing CDH11 shRNA (33 or 34 clonal cells) or siRNA (4A or 6A pooled cell lines). (B) Effect of CDH11 depletion on proliferation of MDA-MB-231 cells measured using crystal violet staining after 5 days. (C) Effect of CDH11 depletion on anchorage-independent colony formation in soft agar. (D) Western blot analysis of CDH11 in PC3 cells CDH11 shRNA, (E) CDH11 knockdown fails to significantly reduce the proliferation of PC3 cells but (F) colony formation is significantly reduced upon CDH11 depletion. (EV, 1B): Empty vector. (Luc) scrambled control. GAPDH was used as a loading control for western blot. Phase image using a 4x objective on a Zeiss inverted microscope. Data are presented as means ± SEM (Student's t-test).

**Figure 3:**
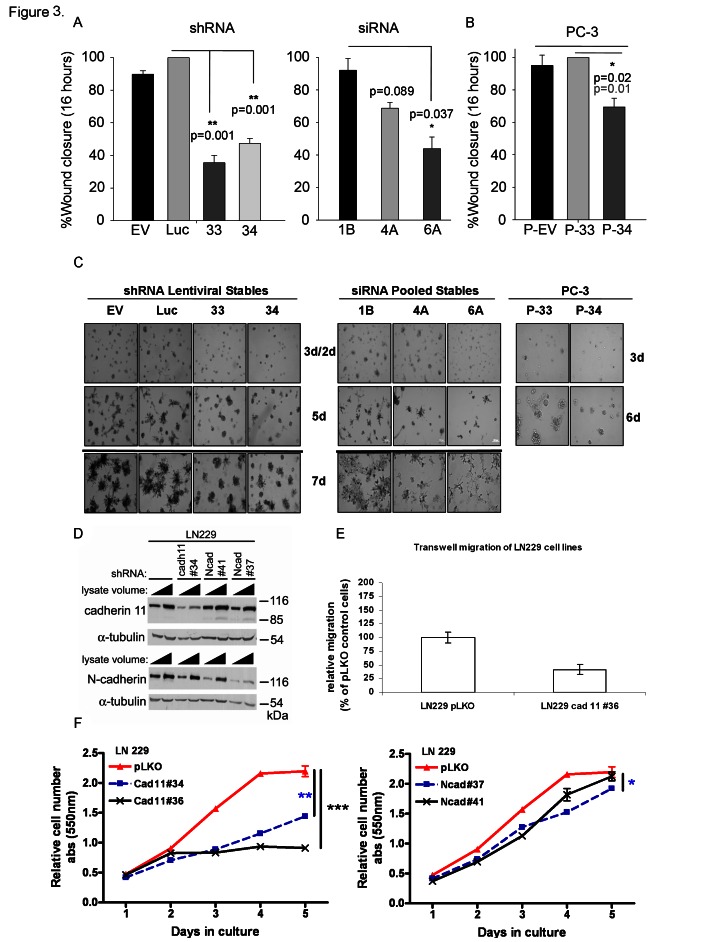
CDH11 knockdown significantly inhibits migration and mediates colony formation of MDA-MB-231 breast and PC-3 prostate cancer cells and is required for the growth and invasion of LN229 glioblastoma cells (A) Effect of CDH11 depletion on the ability of MDA-MB-231 cells and (B) PC3 cells to migrate (x3 separate fields in each well from triplicate wells) 16hr after wounding. (C) Effect of CDH11 depletion on the formation of branched networks on Matrigel™. (D-F) CDH11 or N-cadherin was knocked down in LN229 cells using shRNA. (D) Western blot showing significant reduction in CDH11 and N-cadherin protein, 48 hours post infection with lentivirus containing shRNA. (E) CDH11 knockdown reduces migration and (F) growth of LN229 cells. Note that CDH11 depletion was more effective than N-cadherin knockdown in growth inhibition of LN229 cells. P values determined at day 5: ***p=0.0001, **p=0.0016, *p=0.0425, Ncad#41 vs. pLKO: not significantly different. Columns and bars show the mean and SEM respectively (two-tailed student's t-test).

Reduction of CDH11 in MDA-MB-231 breast cancer cells with siRNA or shRNA (Figure 2A and Supplementary Fig. S3A) significantly decreased growth (Figure 2B and Supplementary Fig. S3B), colony formation (Figure 2C and Supplementary Fig. S3C) and migration (Figure 3A). CDH11 knockdown in PC3 prostate cancer cells (Figure 2D) also resulted in reduced growth, but fell short of significance (Figure 2E). However, PC3 cell colony formation (Figure 2F) and migration (Figure 3B) were significantly inhibited by decreased CDH11. CDH11 knockdown in MDA-MB-231 and PC-3 cells also prevented the formation of branched networks during the first week of culture in Matrigel™ (Figure 3C); but over the following week, CDH11 knockdown cells formed networks that were indistinguishable from controls (data not shown) suggesting that CDH11 is not absolutely necessary for network formation.

Glioblastomas are the most common and invasive brain cancer in humans with poor clinical prognosis. LN229 cells are glioblastoma cell lines that express both mesenchymal cadherins (CDH11 and N-cadherin). Interestingly, the growth and migration of LN229 glioblastoma cells were more sensitive to CDH11 than N-cadherin knockdown (Figure 3D-F).

### The arthritis drug celecoxib (Celebrex™), preferentially inhibits the growth of CDH11 positive basal-like breast cancer cells

**Figure 4:**
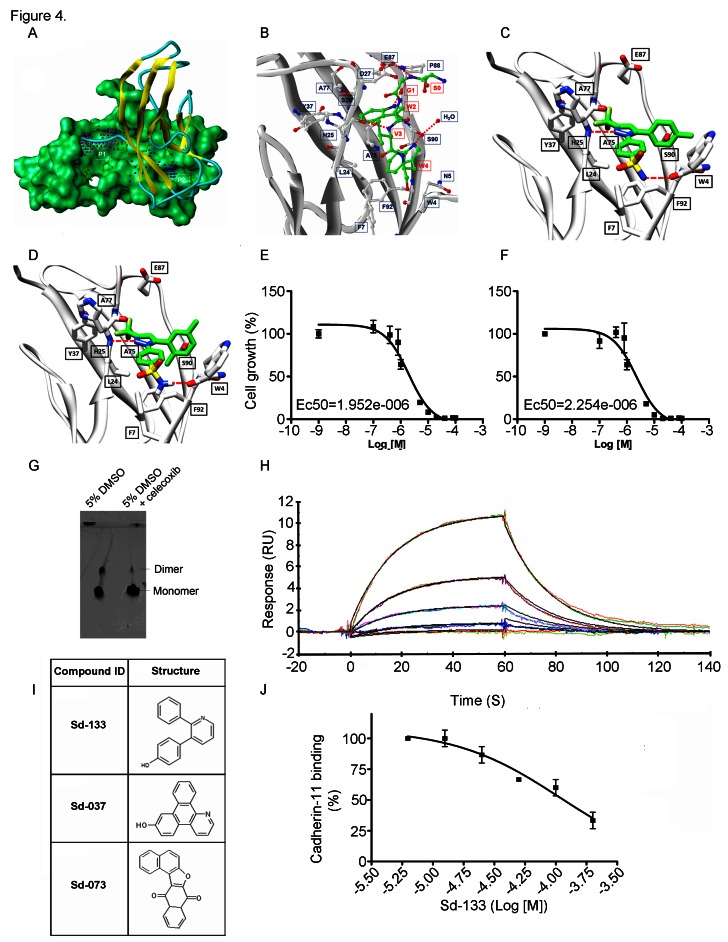
Structural modeling of celecoxib, DMC and other small molecule inhibitors binding to CDH11 and inhibiting the growth of MDA-MB-231 cells. Sd-133 binding capability to CDH11 was validated by SPR (A) EC1 homodimer interface of CDH11 (PDB: 2A4C); one monomer is represented by the Van der Waals molecular surface (green) and the other by a ribbon. P1 is a hydrophobic, concave surface binding to two W residues from the partner EC1 monomer. P2 is a small pocket defined by the EC1 domain itself. Virtual screening was carried out with the residues lining P1 and P2. (B) The EC1 interface with the A strand motif ‘SGWVW’ of the partner EC1 domain (C-atoms-green) contains two W residues. Only residues (black) that make favorable hydrophobic, van der Waals and hydrogen bond contact with the motif (red) are highlighted (H-bonds-dashed lines). (C) 3D structural model of celecoxib and (D) DMC with interactive residue side chains at the tryptophan W-binding pocket (F7, L24, S26, Y37, A75, A77, E87, S90, F92 and W4) are shown in stick rendering, with the carbon atoms of CDH sidechains colored white and the carbon atoms of the inhibitors colored green. The polypeptide backbones are rendered as ribbons. The red broken lines indicate potential intermolecular hydrogen bonds. Oxygen atoms are shown in red, flourine in pale green, nitrogen in blue, and sulfur in yellow. (E) Blocking CDH11 with celecoxib and (F) DMC significantly reduced the proliferation of CDH11 positive MDA-MB-231 as measured using MTS assay. (G) Native gel comparison of cadherin-11 EC1-2 in the absence (left) and presence (right) of celecoxib. Celecoxib was solubilized in DMSO and mixed with purified CDH11 EC1-2 in a 1:1 molar ratio. Note that celecoxib reduces the dimer fraction. (H) Recombinant modified CDH11 protein was immobilized on a Biacore® CM5 Surface by thiol coupling method. Wild type cadherin-11 was injected at various concentrations using Biacore T-200 instrument. Each concentration was injected twice, which showed good binding reproducibility. Colored lines represent real data-points and black lines represent curve fits. (I) 2D structure of the active compounds. (J)Sd-133 competed with CDH11 (ligand) in binding to immobilized CDH11 protein on the surface of the chip.

CDH11 has unique hydrophobic pockets that are potential sites to interfere with cell-cell adhesion (figure 4A) [10]. Recently, using a new proteochemometric computational drug repurposing method we unexpectedly found that the FDA approved drug celecoxib, and DMC a celecoxib analogue without COX2 inhibitory activity had the structural potential to bind CDH11. We used Surface Plasmon Resonance (SPR) [7] and a native gel assay to show direct binding and inhibition of CDH11 dimerization (Figure 4G). The structural models of celecoxib and DMC binding to CDH11 are shown in Figure 4 C and D. Celecoxib and DMC inhibit the growth of CDH11 positive MDA-MB-231, BT549 and Hs578T basal-like breast cancer cells with EC50s in the 1-5 micromolar range but did not affect CDH11 negative MCF7 cells up to 40 micromolar (Figure 4E, F, Supplementary Fig. S4A, B).

### Novel small molecule CDH11 inhibitors specifically inhibit CDH11 mediated growth and migration *in vitro*

**Figure 5:**
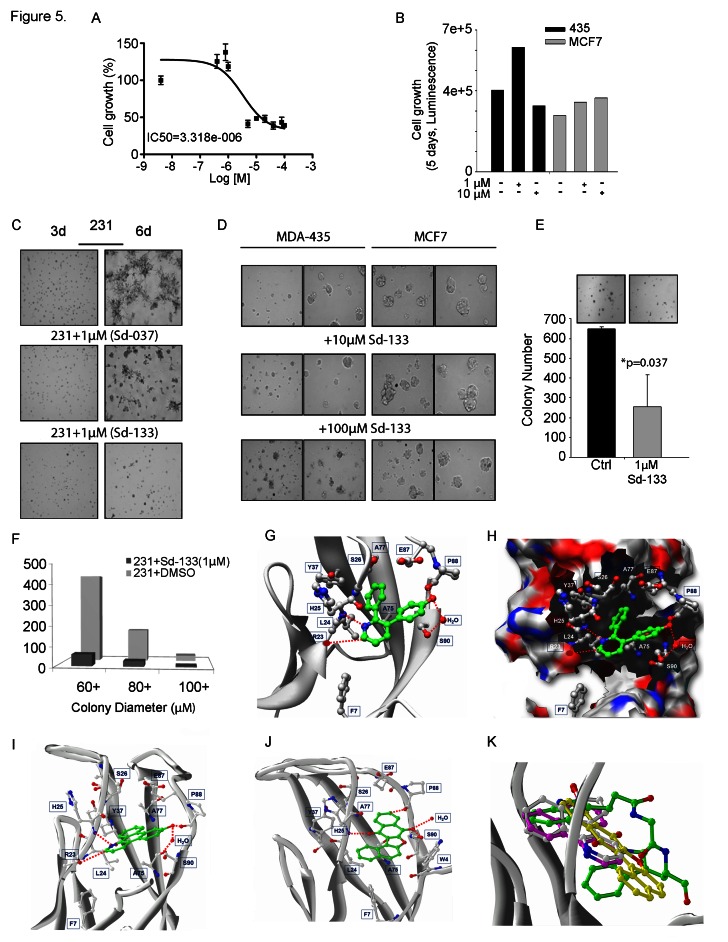
Development of small molecule inhibitors and their effect on CDH11 function-inhibition (A) Blocking CDH11 with sd-133 significantly reduced the proliferation of CDH11 positive MDA-MB-231 as measured by MTS assay. (B) Sd-133 did not inhibit the growth of CDH11-negative MDA-MB-435 melanoma or MCF7 breast cancer cell lines. (C) Sd-037 and Sd-133 significantly impaired MDA-MB-231 outgrowth on Matrigel™. (D) Sd-133 fails to change Matrigel™ morphology of CDH11 negative MDA-MB-435 and MCF7 cells. (E) Effect of sd-133 on anchorage independent colony growth in soft agar. (F) Colony growth at various sizes when MDA-MB-231 cells were treated with Sd-133. (G) Likely binding model of Sd-133. W-binding pocket residues are highlighted (C-atoms-white; H-bonds-red dotted lines). Residues F7, L24, S26, Y37, A75, A77, E87, S90, and F92 contribute hydrophobic interactions and a water mediated interaction with Sd-133. The hydrophobic and H-bond interaction between Sd-133 and CDH11 is similar to that of the two W as seen in (Fig. 5F). (H) Diagram of the concave surface of P1 and P2. W-binding pocket residues are highlighted (C-atoms-white; H-bonds-red dotted lines). Sd-133 is locked into the cavity with H-bond networks on the outside of the concave surface. (I) The H-bond and hydrophobic interactions of Sd-037 and (J) Sd-073 are similar to Sd-133. (K) Superimposition of Cadherin-11 inhibitors Sd-133, Sd-037 and Sd-073 (C-atoms-white) with the W of a partner EC1 monomer motif (C-atoms-green). C: control. Columns and bars show the mean and ESM respectively.

We reasoned that a small molecule that blocks the CDH11 EC1 dimer-formation would inhibit CDH11 function. We used this structure as a basis for molecular simulations to produce pharmacophores designed to block one or both of two adjacent regions (P1 and P2) predicted to be necessary for CDH11 function in cell-cell adhesion. The 29 most promising compounds were obtained and tested. Three compounds: Sd-133, Sd-037, and Sd-073 (Figure 4I) were active in the 1-10uM range attesting to the efficiency of the *in silico* screen (Supplementary Fig. S5). The structure of Sd-133 is the most drug-like, indeed it resembles that of celecoxib, and we chose to move forward with it as our lead compound. Using thiol coupling, we immobilized cysteine-tagged mouse CDH11 (EC1-2 domain) on a SPR CM5 chip and injected wild type CDH11 at different concentrations. SPR demonstrated reproducible dose dependent CDH11 homophilic binding (homodimerization) (Figure 4H). Since, there is simultaneous dimerization occurring both in the injected “analyte” and “ligand” fraction (immobilized CDH11 on the surface) a portion of these molecules will be unavailable for dimerization in this assay and the Kd cannot be precisely calculated using SPR. Equilibrium analytical ultracentrifugation showed that the dissociation constant for CDH11 is 25.2±4.3 micromolar [19;20]. To confirm that Sd-133 binds directly to CDH11, we tested the ability of Sd-133 to compete for CDH11 homotypic binding using SPR. Simultaneous injection of Sd-133 with mouse CDH11 (EC1-2) [19] protein reduced soluble CDH11 binding to immobilized CDH11 on the surface of the chip in a dose dependent manner (Figure 4J). Like celecoxib and DMC, Sd-133 significantly inhibited the growth of all three CDH11 positive cell lines with an EC50 of ~3μM but had little effect on CDH11 negative MCF7 cells (Figure 5A, B, Table 1 and Supplementary Fig. S4C). Sd-133 also inhibited MDA-MB-231 matrigel™ outgrowth at 1μM (Figure 5C) but was inactive on control MDA-MB-435 melanoma cells (express N-cadherin) or MCF7 breast cancer cells that express E and P-cadherin (Figure 5D). In addition, Sd-133 inhibited MDA-MB-231 colony formation (Figure 5E, F). The activity of Sd-133 likely stems from its shape and moderate structural flexibility, which enable it to accommodate and bind tightly to, the W-binding pocket (Figure 5G, H). Though this binding pocket is largely hydrophobic, a network of hydrogen bonds between Sd-133 and R23, H25, P88, S90 confers specificity and rigid binding. Hydrophobic interaction of Sd-133 with F7, L24, S26, Y37, A75, A77, E87, S90, and F92 may also contribute to its action (Figure 5H). Furthermore, the mobility of the water molecule located near S90 (PDB:2A4C) enables this residue to adjust its position to form H-bonds with the inhibitors. Two other inhibitors, Sd-037 and Sd-073, have similar interactions with the W pocket (Figure 5I, J). The water mediated H-bond is observed with all three inhibitors (Figure 5G-J). All three inhibitors compete for W binding and interact with the same residues including the water molecule formed by the two W residues (Figures 4B, 5G-J). Upon superimposition of Sd-133, Sd-037 and Sd-073 within the W pocket, it is clear that the hydrophobic moieties of these three inhibitors occupy the same space as that of hydrophobic W residues (Figure 5K). We tested several W mimics including dindolylmethane (DIM) analogs of the peptide motif ‘SGWVW’, but did not achieve the potency of Sd-133 or celecoxib. Structural modeling and MD simulations indicated that the excessively flexible nature of the peptide mimics impedes the formation of stable interactions in the absence of the rest of the polypeptide backbone.

### *In vitro* and *in-vivo* structure-activity relationship of CDH11 small molecule inhibitors

To understand the relationship of structure to activity of CDH11 inhibitors, we generated several chemical analogues. All compounds were tested by nuclear magnetic resonance (NMR) for purity (see methods). From the ten analogues, only two compounds inhibited the growth of MDA-MB-231 cells with EC50s less than 10μM. Substitution of the R1 methyl group with fluorine completely inactivated the Sd-133 family of compounds, as did any modification of the R2 methyl group. Roughly equivalent activities were seen when R1 was a methyl, methoxy or hydroxyl group. Subsequently, we tested the effect of the compounds that passed our initial screen on the BT549 and Hs578T cells. The active analogues were selectively effective on CDH11 positive cell lines with EC50s in the low μM range and did not affect CDH11 negative MCF7 cells up to 40 μM (Supplementary Fig. S4D, E and Table 1).

**Table 1:** Structure activity relationship of different Sd-133 analogues, celecoxib and DMC Various substituent (R1 and R2) are shown along with growth inhibition EC50s for three cadherin-11 positive cell lines. NA, Non-applicable: compounds with no significant growth inhibition in MDA-MB-231 cells (EC50 higher than 10 μM shown in red) were omitted for further analysis in other cell lines

Structure	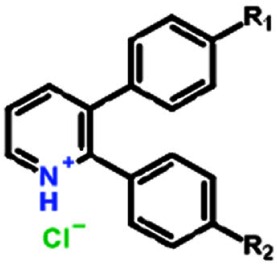					
Compound	Systematic (IUPAC) Name	R1	R2	EC50 (MDA-231)	EC50 (BT549)	EC50 (HS578-t)
Sd133-0001A	3-(4-methoxyphenyl)-2-phenyl-pyridin-1-ium chloride	OCH3	H	1.27E-06	1.57E-06	1.95E-06
Sd133-0002A	3-(4-fluorophenyl)-2-phenyl-pyridin-1-ium chloride	F	H	>10E-06	NA	NA
Sd133-0003A	4-(2-phenylpyridin-1-ium-3-yl)phenol chloride [The hydrochloride salt of SD-CAD11-133]	OH	H	4.53E-06	2.25E-06	1.47E-06
Sd133-0004A	3-(4-fluorophenyl)-2-(4-methoxyphenyl)pyridin-1-ium chloride	F	OCH3	>10E-06	NA	NA
Sd133-0005A	2,3-bis(4-methoxyphenyl)pyridin-1-ium chloride	OCH3	OCH3	>10E-06	NA	NA
Sd133-0008A	2-(4-methoxyphenyl)-3-phenyl-pyridin-1-ium chloride	H	OCH3	>10E-06	NA	NA
Structure	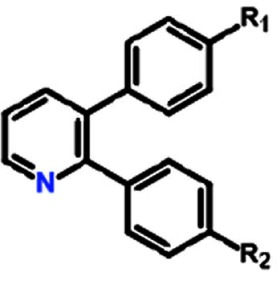					
Compound	Systematic (IUPAC) Name	R1	R2	EC50 (MDA-231)	EC50 (BT549)	EC50 (HS578-t)
Sd-133	4-(2-phenyl-3-pyridyl)phenol	OH	H	3.32E-06	3.07E-06	2.77E-06
Sd133-0006	4-(3-phenyl-2-pyridyl)phenol	H	OH	>10E-06	NA	NA
Sd133-0007	4-[3-(4-methoxyphenyl)-2-pyridyl]phenol	OCH3	OH	>10E-06	NA	NA
Structure	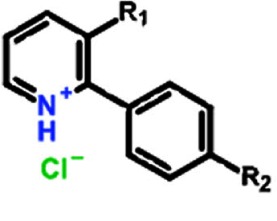					
Compound	Systematic (IUPAC) Name	R1	R2	EC50 (MDA-231)	EC50 (BT549)	EC50 (HS578-t)
SD133-0009A	3-bromo-2-phenyl-pyridin-1-ium chloride	Br	H	>10E-06	NA	NA
SD133-0010A	3-bromo-2-(4-methoxyphenyl)pyridin-1-ium chloride	Br	OCH3	>10E-06	NA	NA
Structure	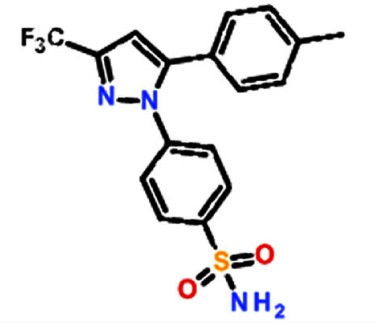					
Compound	Systematic (IUPAC) Name	EC50 (MDA-231)			EC50 (BT549)	EC50 (HS578-t)
Celecoxib	4-[5-(4-methylphenyl)-3-(trifluoromethyl) pyrazol-1-yl]benzenesulfonamide	1.95E-06			4.07E-06	1.27E-06
Structure	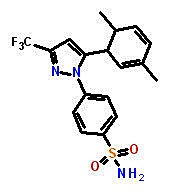					
Compound	Systematic (IUPAC) Name	EC50 (MDA-231)			EC50 (BT549)	EC50 (HS578-t)
Dimethylcelecoxib	4-[5-(2,5-Dimethylphenyl)-3-(trifluoromethyl)-1H-pyrazol-1-yl]benzenesulfonamide	2.25E-06			4.43E-06	1.42E-06

**Figure 6:**
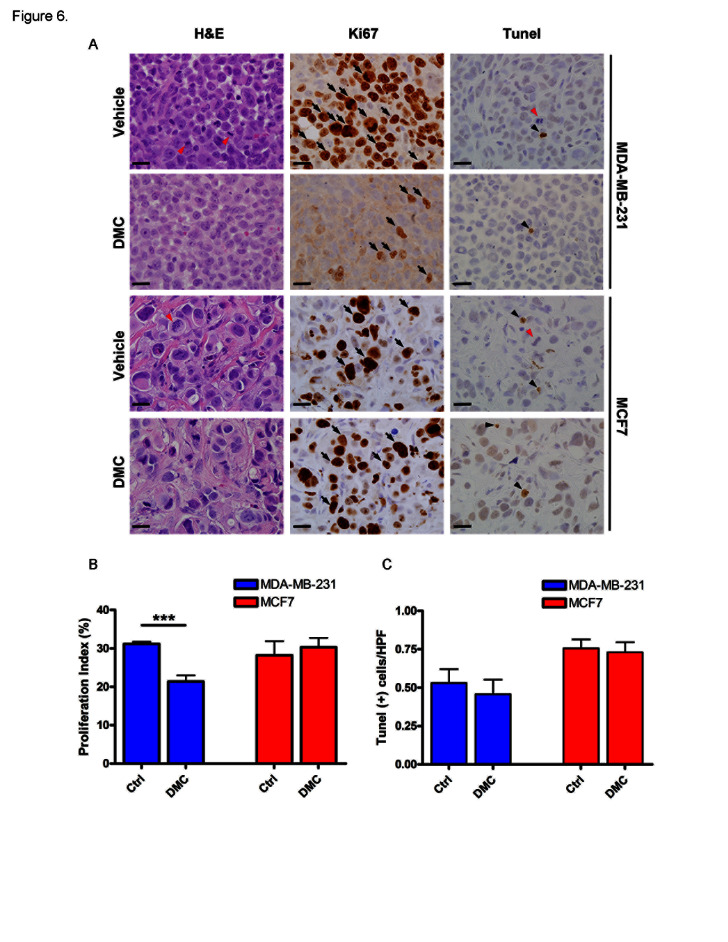
4-[5-(2,5-Dimethylphenyl)-3-(trifluoromethyl)-1H-pyrazol-1-yl]benzene sulfonamide (DMC) significantly reduced the proliferation of MDA-231 cells but had no effect on CDH11-negative MCF7 cells in vivo Nude mice were implanted with 6x10^6^ MDA231 (N=4/cohort) or MCF7 cells (N=3/cohort) in the mammary fat pad. Once the tumors became palpable, animals were treated with (150 mg/kg/day) of DMC or vehicle for 48 hours and euthanized 2 hours post final treatment. (A) Representative ki67 immunostaining (proliferation marker), TUNEL (apoptosis marker) and corresponding H&E sections from xenografts. (B) DMC significantly lowered the proliferation rate of MDA231 transplanted cells but not MCF7 cells. (C) DMC did not have any significant effect on the apoptosis rate of cancer cells. Arrows indicate representative proliferative cells. Black arrow-heads demonstrate apoptotic cells and red arrow-heads indicate mitotic-figures. Scale bar= 20 micrometers. Columns and bars show the mean and ESM respectively (student's t-test). ***p=0.0006

We chose DMC to test in animals bearing CDH11 expressing tumors. DMC has structural and EC-50 similarity to our lead compound sd-133, does not inhibit COX2 and has previously been used in animals [21]. As early as 50 hours following administration, the proliferation rate of CDH11 positive MDA-231 tumors was significantly reduced (P=0.0006) whereas CDH11 negative MCF7 tumors were unaffected (Figure 6A, B). No differences were observed in apoptosis, suggesting that DMC was not toxic at this dosage (Figure 6C)

## DISCUSSION

The work presented here confirms the association of CDH11 with a number of malignancies and demonstrates that CDH11 is an important driver of certain poor prognosis cancers, as it is in RA. We further show that CDH11 can be directly targeted in tumor cells using CDH11 antibodies and repositioned drugs such as celecoxib that are effective in RA. Taken together with our discovery of novel small molecule inhibitors there is a strong possibility that therapeutic options for CDH11-dependent malignancies and inflammatory diseases may be rapidly developed and tested in humans. It is plausible that CDH11 expression in breast cancer is a component of the EMT that allows cells to progress and metastasize [22]. CDH11 is expressed in breast cell lines characterized as Basal B, as well as poor prognosis malignancies including glioblastoma multiforme for which no effective treatment exists [5;9]. These associations suggest that CDH11 is expressed in highly aggressive tumors resistant to treatment. Breast tumor stroma contains mesenchymal stem cells that express factors vital for malignant progression [15]. CDH11 is expressed by mesenchymal stem cells prior to differentiation into adipose or muscle tissues, continue through their differentiation into chondrocytes and osteoblasts and is observed in the stromal component of DCIS lesions [23;24]. CDH11 was also mostly limited to the stroma of the ILCs. Consequently, although our data demonstrates that stromal CDH11 is not exclusively responsible for changes in tumor growth at least in breast cancer, CDH11 expressing cells in the stroma almost certainly contribute to tumorigenesis, perhaps even in CDH11-negative tumors.

MDA-MB-231 cells preferentially metastasize to the skeleton following intracardiac injection in nude mice [8]. CDH11 may facilitate bone metastasis by mediating direct interaction with osteoblasts [25]. However, it is necessary to distinguish anti-growth effects of CDH11 depletion from anti-metastatic properties. In our studies cell proliferation and tumor growth were inhibited upon CDH11 depletion in MDA-MB-231 cells suggesting that a portion of the “metastatic” potential may be a result of alterations in cell proliferation. The current work also demonstrates the critical role that CDH11 plays in tumorigenesis of aggressive, basal or mesenchymal-like breast cancer. We propose that cells, which have undergone EMT, respond unfavorably to depletion of CDH11 because it is essential for their growth and progression. Similarly, synoviocytes depend on CDH11, for migration, invasion and growth [6]. Our analysis of all published human breast cancer microarray datasets as well as our immunohistochemical studies showed that increased CDH11 is an early event in breast cancer progression. Breast cancer patients may also shed CDH11 and/or CDH11-expressing cells into the circulation, which could potentially be utilized as a biomarker and/or companion diagnostic [26].

Although our microarray meta-analyses did not show a relationship between CDH11 (or N-cadherin) to prostate cancer (Supplementary Fig. S1A) the datasets we examined, did not indicate, and likely do not include androgen resistant forms of the disease in which both N-cadherin and CDH11 are elevated [27;28]. Indeed, significant reduction of growth and cell migration occurred upon CDH11 knockdown in PC3 prostate cancer cells [29]. We did not observe such a strong growth effect but noted significant reduction in cell migration and delayed Matrigel™ outgrowth.

CDH11 is a major target in RA, an inflammatory disease frequently compared with cancer. CDH11 controls the synovial response in RA and targeting CDH11 either by knockout or with antibodies reduced RA in mouse models [6]. In this study we show that the same CDH11 antibodies significantly inhibited the growth of mesenchymal/basal-like MDA-MB-231 xenografts. The cycloxygenase-2 (COX-2) selective NSAIDS such as celecoxib (Celebrex™) and the now discontinued rofecoxib (Vioxx™) are treatments of for patients with RA. Celecoxib also has well known anti-cancer properties [30-33] and RA patients treated with celecoxib or rofecoxib have a lower risk of breast, prostate and colorectal cancers [34;35]. However, although there is no doubt that celecoxib is an excellent inhibitor of COX-2 there is much debate over its COX-2 independent activities and if these could play a role in its anti-inflammatory and anti-cancer effects [36-39]. Recently, drug-repositioning has come into the spot-light as a significant time saving and cost effective alternative to traditional drug-discovery methods [7;40]. Our recent computational drug repurposing screens indicated that celecoxib is likely to interact with targets other than COX-2 and in a completely serendipitous observation, predicted that celecoxib is likely to interact with the CDH11 tryptophan pocket [7]. In the present study we confirmed the CDH11 inhibitory activity of celecoxib and its inactive (wrt COX2-inhibition) analogue DMC. Previous studies show that celecoxib preferentially inhibits the growth of xenografts of cells we now know to be CDH11 expressors such as MDA-231 cells and gliomas [41;42]. In addition celecoxib induces apoptosis in CDH11 positive synovial fibroblasts in a COX-2 independent manner and DMC inhibits glioma growth in animals [21;43]. Here we show a significant reduction in proliferation of MDA-231 transplanted cells using DMC. Taken together these data suggest that, in addition to other suggested celecoxib targets such as survivin, the ER stress response and PDK1, CDH11 may play a role in mediating the COX-2 independent effects of the Celebrex™ family of anti-inflammatories in cancer and in RA [42;44].

Although the binding mode of celecoxib and DMC to the CDH11 tryptophan-binding pocket is slightly different from the CDH11 inhibitors specifically designed to do so (Figure 4C, D), all the effective CDH11 inhibitors compete for hydrogen bonds with the H25 backbone. The volume and topography of the pockets in the tryptophan site (Figure 4 C, D) may provide an opportunity to modify and optimize CDH11 inhibitors for shape, size, and polarity to maximize interaction and potency.

CDH11 over-expression in a subset of DCIS indicates it as an early event in breast cancer development. Although invasiveness of a tumor is likely determined in early pre-malignant phases [45] not all DCIS lesions develop into invasive ductal carcinomas [46]. As not all DCIS lesions are CDH11 positive and high expression occurs in comedo type DCIS, a subclass with greater risk of recurrence, CDH11 positive lesions may be more likely to develop into invasive breast cancer [47]. Our data indicates that CDH11 is an important factor in malignant progression and is a promising therapeutic target in poor prognosis breast cancers and other CDH11 expressing malignancies such as glioblastoma, and perhaps androgen-independent prostate cancer. We also introduce an antibody and small molecule inhibitors of CDH11 as well as an FDA approved drug that are able to inhibit its function.

## METHODS

### Cell Culture and Generation of Stable Cell Lines

MDA-MB-231, BT-549, HS578, MDA-MD-435, MCF7 and PC-3 cancer cell lines (ATCC), and all stable cell lines generated were maintained in DMEM (Invitrogen) supplemented with 5-10% fetal bovine serum (FBS) as previously described [5]. siRNA vectors were synthesized using the Silencer™ siRNA Construction Kit (Ambion) and co-transfected with hygromycin B-resistant vector or vector alone using Fugene (Roche Diagnostics). Stable clones were selected using 1mg/mL hygromycin B and maintained at 0.5 mg/mL. siRNA stable lines were created using templates with T7 promoter sequences at 3’ end and an AA 5’ overhang (IDT Inc.), using anti-sense (5’-*AACAGCGTGGATGTCGATGACCCTGTCTC*-3’) and sense (5’-*AAGTCATCGACATCCACGCTGCCTGTCTC*-3’) sequences to target CDH11. shRNA stables were created using MISSION® shRNA lentiviral transduction particles (Sigma-Aldrich) directed against human CDH11. Single cell clones targeting two separate shRNA sequences of the same CDH11 region were used to infect MDA-MB-231 breast and PC-3 prostate cancer cells. Clones were selected in 15μg/mL puromycin and maintained at 10μg/mL.

### Western blot, immunocytochemistry and immunohistochemistry

Western blots and immunocytochemistry were carried out as described previously [5]. For Ki67 stained cells, 10 random high power fields representing the whole tumor area were selected and 1687±33 cells/tumor (MDA-231, n=4/group) and 1405±59 cells/tumor (MCF7, n=3/group) were counted. The proliferation index was measured as the number of positive cells/total cells counted x100. Since the rate of apoptosis is much lower than proliferation, we wanted to avoid any potential bias due to field selection; therefore the number of TUNEL positive cells in the whole tumor section was counted and the apoptosis index was calculated as the number of positive cells/the number of fields per sample.

### MTS cell proliferation/Survival assay

As cadherin-11 expression is increased with cell density [48], we determined the maximum cell density for each cell line that would allow us to be within linear range of assay detection up to 96 hours post seeding. Based on those results we used 4000 (Hs578t) or 8000 (all other cell lines) cells/well in 96 well plates for MTS proliferation assays. Cells were initially treated at the time of seeding and medium + compounds replenished at 24, 48 and 72 hours post seeding. 96 hours post-seeding, medium was replaced with fresh serum-free medium followed by MTS reagent. Absorbance was read 2 hours post MTS addition at 490 nm.

### *In-vivo* studies to assess the effect of CDH11 knockdown, CDH11 antibody and CDH11 inhibitors

6-7 week old female athymic nude mice (Harlan research laboratory) were inoculated with approximately 2 x10^6^ cells in the mammary fat pad. For growth inhibition studies, mice were treated i.p. initially with 0.5 mg 13C2 (anti-CDH11) or control IgG followed by 0.1 mg for subsequent injections (x3/wk) for one month. This antibody was generated by immunizing CDH11 deficient mice with purified CDH11 EC(1-5)-mouse IgG2a FC fusion protein followed by generation of hybridomas and validated to be specific against cadherin-11[6]. We used the (π/6)xLxWxH formula to calculate tumor volume. To study the effect of antibody therapy on inhibition of established tumors, mice were left untreated post inoculation until tumor size reached approximately 50 mm^3^ then were treated with 20 mg/kg (x2/wk). For xenografts of RNAi or shRNA expressing MDA-MB-231 cells, 1-2 ×10^6^ stable cells were injected s.c. into 1 of 4 ventral side mammary fat pads such that both control and RNAi-expressing cells were injected in each animal. Tumor volume was calculated using the formula D1×D2×D3 (D1=length, D2=width, D3=depth of tumor) using calipers, ×2/week. Experiments were repeated ×3 for all cell lines. To study the effects of CDH11 inhibitors on tumor cell proliferation in vivo we inoculated nude mice with MDA-231 or MCF7 cells. Once the tumors became palpable, animals were treated with 150mg/kg/day of DMC by oral gavage.

### Surface Plasmon Resonance

A Biacore T200 instrument and CM5 sensor chip was utilized. Mouse EC 1-2 C-terminally cysteine-tagged CDH11 protein [49] was immobilized on flow cell (FC) 4 in HEPES Buffered Saline (10 mM Hepes, pH 7.4; and 150 mM NaCl, 3mM CaCl_2_) using thiol-coupling kit according to the manufacture's protocol, resulting in immobilization level of 4673 response units (RU). FC3 was used as a reference for background noise elimination. For homophilic dimerization experiments 2.5, 1.25, 0.625, 0.313 and 0.156 micromolar wild type CDH11 [49] were injected twice and CDH11–CDH11 binding was measured. For competition experiments, Sd-133 at concentrations of 200, 100, 50, 25, 12.5 and 6.25 micromolar were co-injected with 2.5 micromolar intact EC1-2 CDH11 protein (buffer: 10mM Hepes, 150mM NaCl, 3mM CaCl_2,_ 1% DMSO). Each injection was repeated ×2 for 60 s.

### Generation and synthesis of SD-133 analogues

To generate Sd-133 analogues, we first synthesized an intermediate structure as a base for all other active pharmacophores and later different chemical groups are added. Regiochemistry of the intermediate compound was determined by as described by Karig et al [50].

### Intermediate 1

#### 3-Bromo-2-Phenylpyridine

**Figure:**
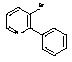


Aqueous potassium carbonate (2M, degassed, 6 mL) was added to a stirred solution of 2,3-dibromopyridine (260 mg; 1.1 mmol), benzeneboronic acid (137 mg; 1.12 mmol) and triphenylphosphine (20 mg; 0.07 mmol) in THF (oxygen free; 4 mL). Tetrakis(triphenylphosphine)palladium(0) (40 mg; 0.035 mmol) was added and the mixture heated under reflux for 18 hrs. The mixture was partitioned between dichloromethane and water, separated and the aqueous solution extracted with dichloromethane. The combined dichloromethane solutions were washed with brine, dried (MgSO_4_) and evaporated to give the crude product, which was used in subsequent steps without further purification.

^1^H NMR (400MHz, CDCl_3_) d8.63 (1H, dd, J = 4.8, 1.2), 8.00 (1H, dd, J = 8.0, 1.2), 7.70-7.30 (>5H, m, Ph + residual Ph_3_P signals) and 7.15 (1H, dd, J = 8.0, 4.8).

NMR showed this material to be impure product, from which the product regiochemistry was determined to be 3-bromo-2-phenylpyridine, by reference to the NMR of the alternate 2-bromo-3-phenyl- isomer; Product 8b shows pyridyl signals at d8.37 (1H, dd, J = 4.9, 2.0), 7.62 (1H, dd, 7.6, 2.0) and 7.33 (1H, dd, J = 7.6, 4.9)].

#### Sd133-0001A

#### 3-(4-Methoxyphenyl)-2-phenylpyridinium Chloride

**Figure:**
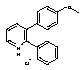


Aqueous potassium carbonate (2M, degassed, 1.5 mL) was added to a stirred solution of 3-bromo-2-phenylpyridine (Intermediate 1; 45mg; assumed 0.19 mmol) and 4-methoxyphenylboronic acid (60 mg; 0.39 mmol) in THF (oxygen free; 1 mL). Tetrakis(triphenylphosphine)palladium(0) (30 mg; 0.026 mmol) was added and the mixture was heated under reflux for 18 hrs before being partitioned between dichloromethane and water. The aqueous solution was extracted with dichloromethane and the combined organic solutions washed with brine, dried (MgSO_4_) and evaporated. The resulting crude mixture was chromatographed on silica gel using a gradient of 5%-15% v/v ethyl acetate in hexane as solvent to afford the desired product as the free base (45 mg).

^1^H NMR (400MHz, CDCl_3_) d8.66 (1H, dd, J = 4.8, 1.6), 7.70 (1H, dd, J = 8.0, 1.6), 7.40-7.35 (2H, m), 7.30 (1H, dd, J = 8.0, 4.8), 7.25-7.20 (3H, m), 7.10 (2H, d, J = 9.6), 6.80 (2H, d, J = 9.6) and 3.79 (3H, s).

This material was dissolved in diethyl ether (5 mL) and hydrogen chloride in dioxane (4M; 1 mL) was added. The mixture was concentrated to a solid which was suspended in diethyl ether, filtered and washed with hexane, then dried *in vacuo* to afford the hydrochloride salt as a white solid (36 mg).

^1^H NMR (400MHz, CDCl_3_) d8.95 (1H, br d, J = 4.8), 8.34 (1H, br d, J = 7.6), 7.86 (1H, m), 7.57-7.38 (5H, m), 7.09 (2H, d, J = 8.6), 6.87 (2H, d, J = 8.6) and 3.77 (3H, s). Exchangeable NH^+^ proton not seen.

### Sd133-0003A (Free base) and Sd-133 (Hydrochloride Salt)

#### 4-(2-phenyl-3-pyridyl)phenol

#### 4-(2-Phenylpyridin-1-ium-3-yl)phenol Chloride

**Figure:**
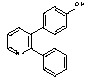

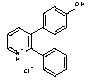


In an analogous manner to that described for *Sd133-0001A*, a mixture of aqueous potassium carbonate (2M, degassed, 1.5 mL), 3-bromo-2-phenylpyridine (Intermediate 1; 85mg), 4-(*tert*-butyldimethylsilyloxy)phenylboronic acid (184 mg), tetrakis(triphenylphosphine)palladium(0) (40 mg) and THF (oxygen free; 1 mL) was reacted for 18 hrs under reflux, subjected to aqueous workup and chromatographed to give impure O-silyl product. This crude product (60 mg) was dissolved in THF (anhydrous, 2 mL) and cooled to 0°C and stirred during the portion-wise addition of a solution of tetrabutylammonium fluoride (1M in THF, 0.3 mL, assumed 1.5 eq). The mixture was allowed to warm to room temperature and stirred for a further 2 hrs before being diluted with ethyl acetate (10 mL) and partitioned with water (10 mL). The phases were separated and the organic solution was washed with water (2 × 10 mL), then the combined aqueous solutions were back-extracted with ethyl acetate (15 mL). The combined organic solutions were dried (Na_2_SO_4_), filtered and evaporated to afford the crude product (70 mg). This was purified by chromatography on silica gel using a gradient of 10%-40% v/v ethyl acetate in hexane to give the free base as an off white solid (25 mg).

^1^H NMR (400MHz, CDCl_3_) d8.66 (1H, dd, J = 4.8, 1.7), 7.70 (1H, dd, J = 7.7, 1.7), 7.45- 7.20 (6H, m), 7.03 (2H, d, J = 8.7), 6.70 (2H, d, J = 8.7) and 5.00 (1H, br).

The free base was converted to the hydrochloride salt in a manner analogous to that described for *Sd133-000A1*.

^1^H NMR (400MHz, CDCl_3_+d_6_-DMSO) d8.85 (1H, br d, J = 5.2), 8.36 (1H, br d, J = 7.6), 7.89 (1H, m), 7.55-7.30 (5H, br m), 6.98 (2H, d, J = 8.5) and 6.84 (2H, d, J = 8.5). Exchangeable protons not seen.

### Soft Agar Assays

5,000 cells were plated in 0.3% agar layered on top of 0.6% agar in 35-mm^2^ plates. After 2 weeks, the colonies were counted in an Omnicon 3600 automated colony counter (BioLogics, Inc.) and visualized using a SMZ-1500 stereoscope (Nikon). These experiments were all carried out in 5% serum.

### Wound Healing and Matrigel™ Outgrowth Assays

Cells were grown to confluency in DMEM +5% FBS, and vertical scrape wounds were made in each well with a 10μL pipette tip. Images were recorded immediately following scraping using a Nikon Eclipse TE-300 inverted microscope with motorized stage and CO_2_-regulated chamber. Phase contrast images were taken every 1 h (10x objective). For Matrigel™ outgrowth assays, cells were plated in duplicate in 12-well glass-bottom dishes (MatTek, Ashland, MA) coated with 150μl of Matrigel™ (BD Biosciences, San Jose, CA). Cells (5,000 cells/100 μl medium) were plated atop the Matrigel™ layer, set 30 min at 37°C, then 1ml growth medium was gently added to each well. Growth was visualized using a 5x objective on an AH2 Vanox inverted microscope (Olympus).

### *In-vivo* experiments to assess the effect of small molecule inhibitors

8-9 weeks old female athymic nude mice (Harlan research laboratory) were inoculated with approximately 6 x10^6^ MDA-231 or MCF7 cells in the mammary fat pad. Once the tumors were palpable, animals were treated with DMC or vehicle alone (50% ETOH, 0.04% methylcellulose and 0.002% Tween-20) at a dose of 150 mg/kg/day (animals were treated twice a day with half of daily dose intake for each treatment episode)) through oral gavage for 48 hours. Mice were euthanized at 50 hours (2 hours post final treatment) and tumors dissected, fixed in formalin and submitted for immunostaining.

## SUPPLEMENTARY FIGURES


