# Supplementary material for: Cadherin-11 in poor prognosis malignancies and rheumatoid arthritis: common target, common therapies
Source: Oncotarget. 2013 Nov 15;5(6):1458–74. doi: 10.18632/oncotarget.1538 (PMC4039224; doi:10.18632/oncotarget.1538)
Supplement: Supplementary file 1 [file oncotarget-05-1458-s001.doc]

**Supplementary Figure S1.**

**
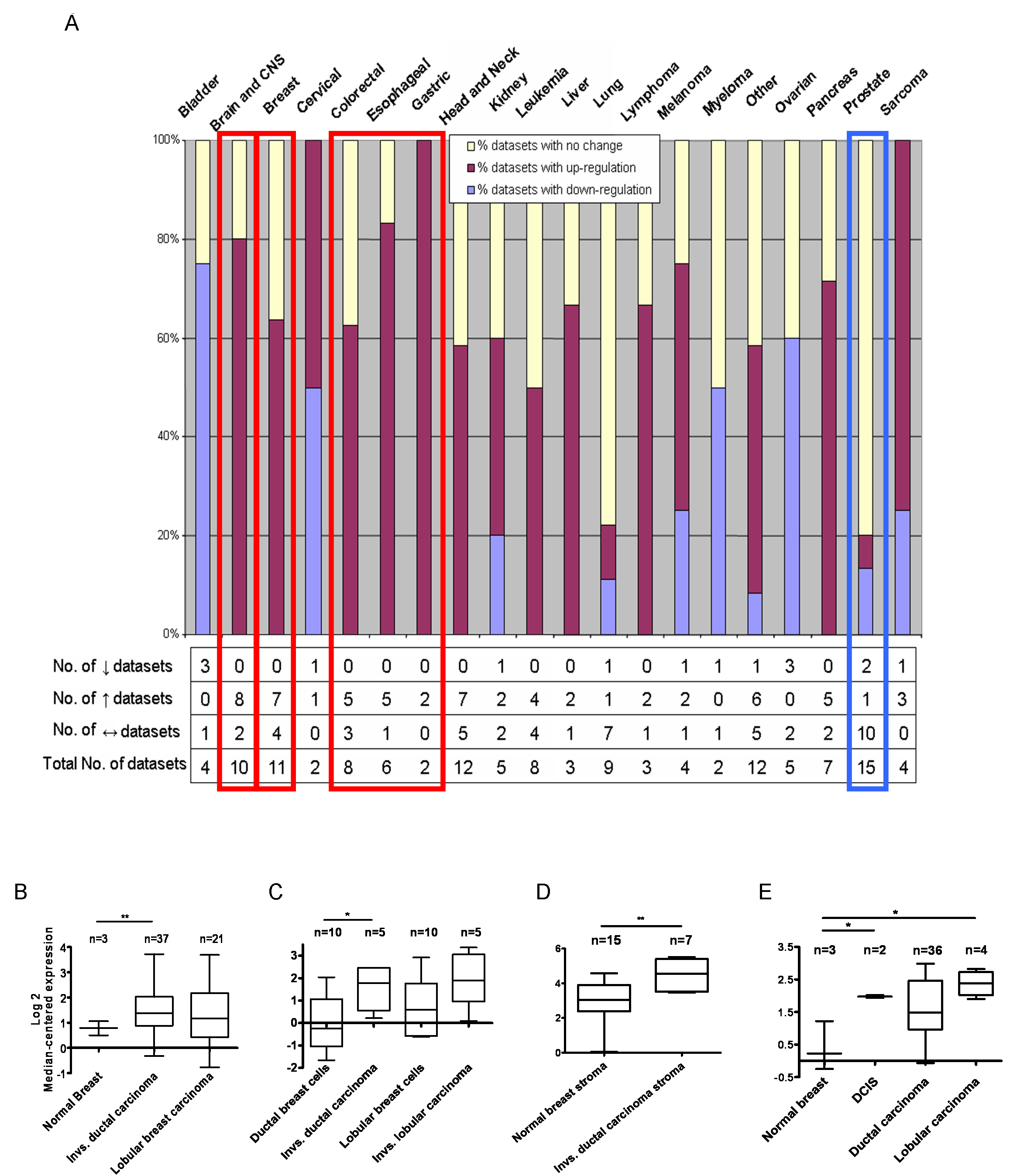
**

**Supplementary Figure S2.**

**
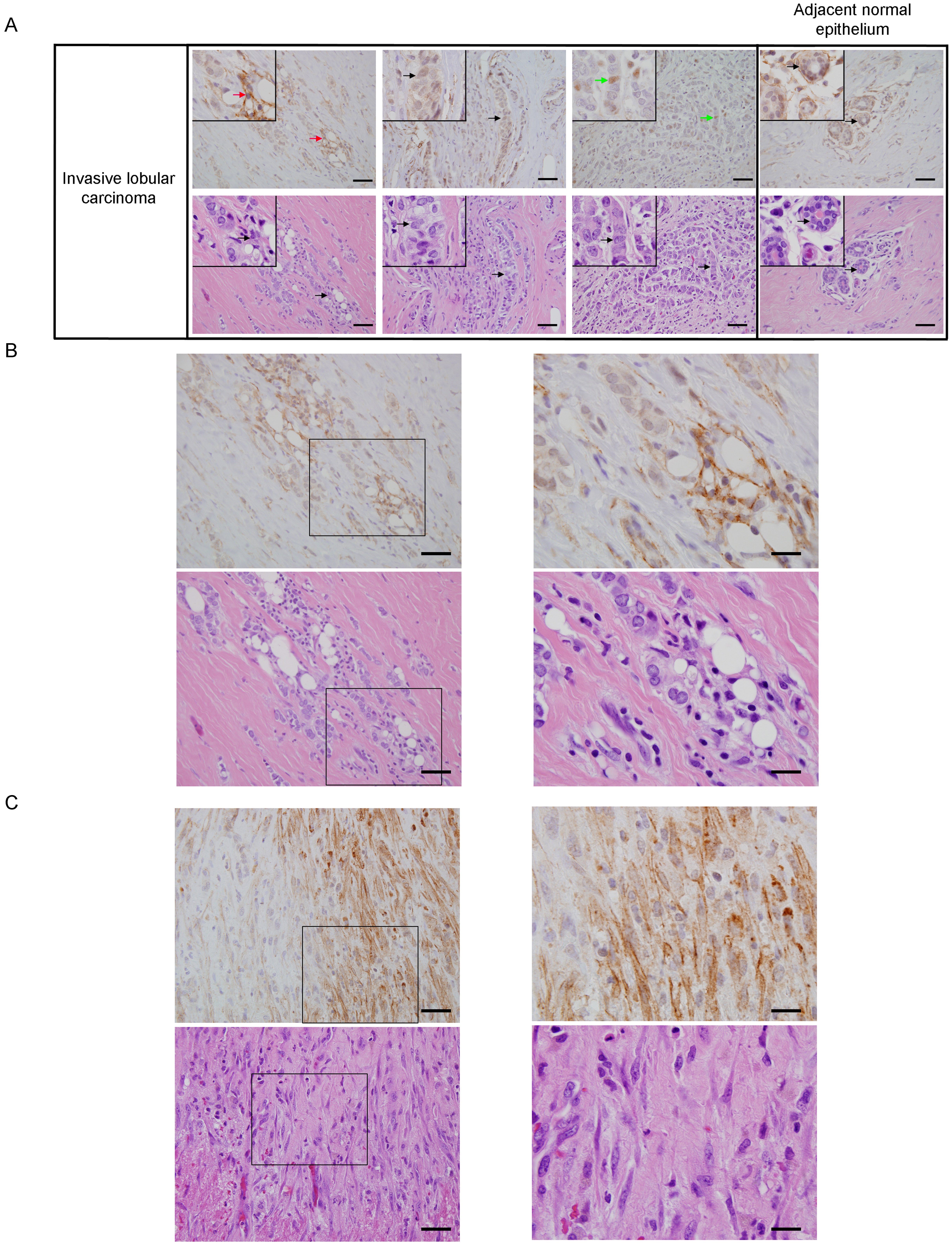
**

**Supplementary Figure S3.**


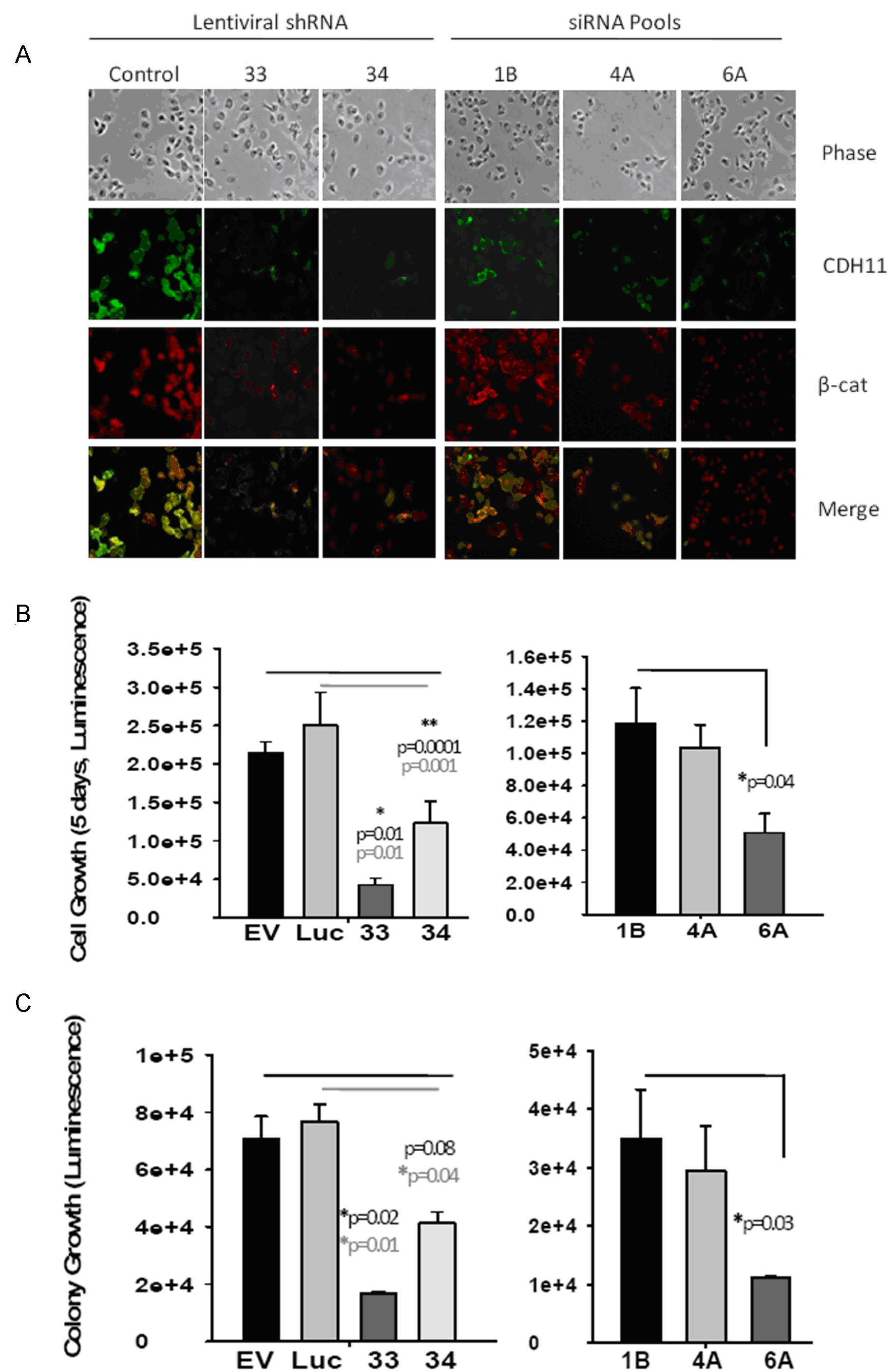


**Supplementary Figure S4.**

**
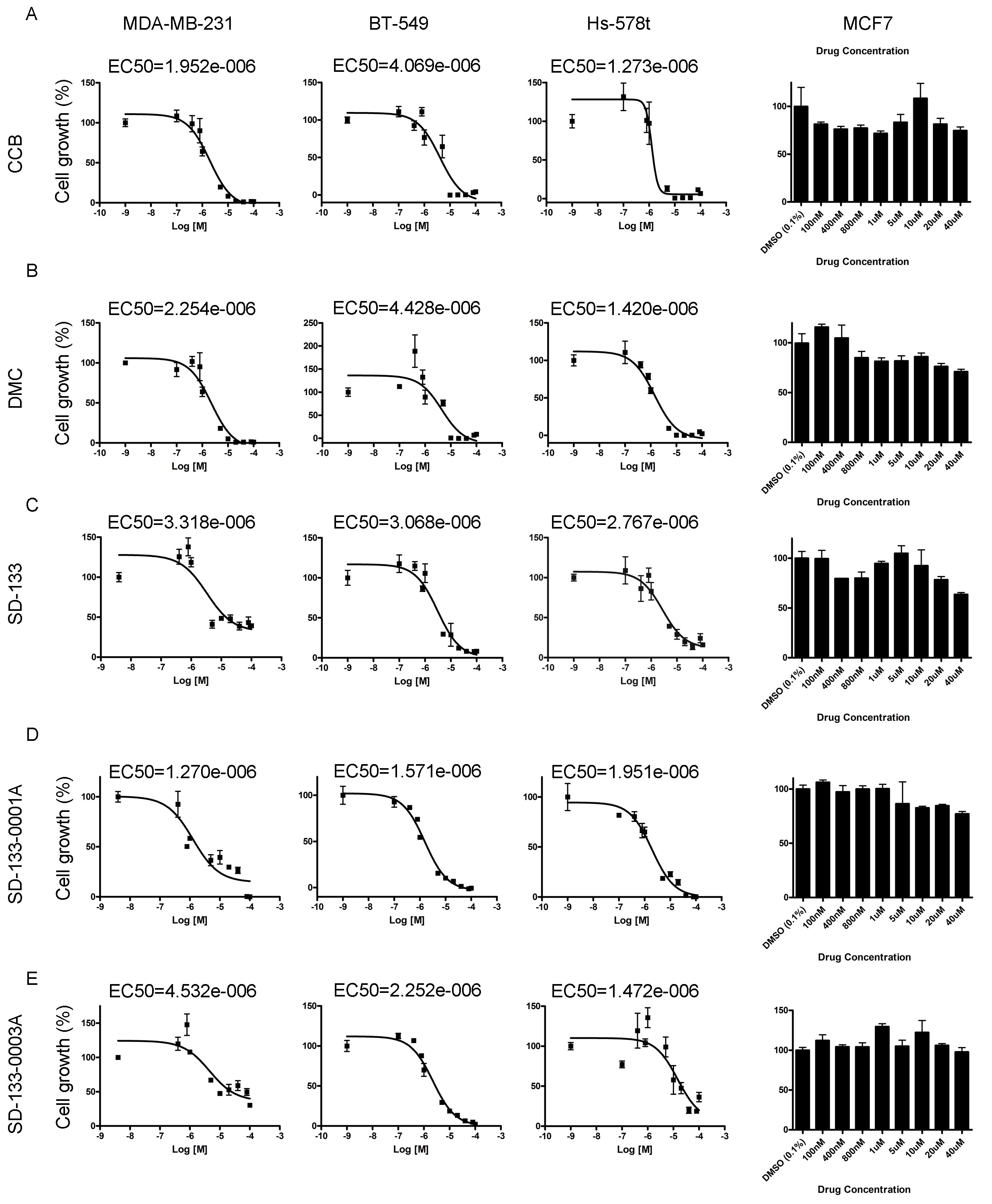
**

**Supplementary Figure S5.**


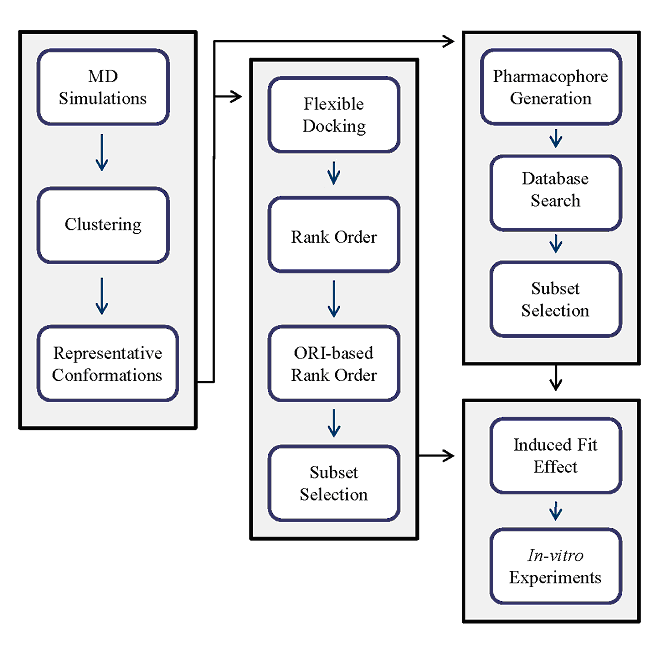


**Supplementary Figure S1.**  **Cadherin11 transcript pattern of all publicly available cancers and corresponding normal tissues and its over-expression in ductal carcinoma in situ and invasive breast carcinoma. (Oncomine^TM^**). (A) Some cancers demonstrate both up-regulation and down-regulation in different datasets (eg. prostate, kidney, and lung). Studies were categorized by cancer type according to Oncomine grouping and compared to corresponding normal tissue. Values are shown as percentage of datasets with and without a significant change in cadherin-11 expression. Red rectangles highlight cancers in which most datasets over-express cadherin-11; the blue rectangle represents cancers in which cadherin-11 mRNA expression can go up or down in different datasets. Analysis thresholds were set at P<0.01, Fold change >1.5 including and all gene ranks included. (B-C) Cadherin-11 expression is significantly increased in the whole invasive breast carcinomas (13-14) as well as (D) stromal compartment of the tumor (15) when compared to corresponding normal tissue. (E) Significant up-regulation is an early event in ductal carcinoma in-situ (16). The fold changes in the expression level of cadherin 11 and p values (Welch’s t-test) were as follows: (B) Invasive ductal carcinoma vs. normal breast: p=0.0087, FC: 1.642, lobular breast carcinoma vs. normal breast: p=0.0539, (fold change) FC: 1.448 (13), (C) Invasive ductal carcinoma vs. normal adjacent ductal breast cells: p=0.0115, FC: 3.009, invasive lobular carcinoma vs. normal adjacent lobular breast cells: p=0.06, FC:2.280 (14), (D) Invasive ductal carcinoma stroma vs. normal breast stroma: p=0.0033, FC: 2.753 (15), (E) Ductal carcinoma in situ vs. normal breast: p=0.0343, FC: 2.980, lobular carcinoma vs. normal breast: p=0.0276, FC: 3.927 (16), ↑: datasets with significant CDH-11 upregulation, ↓: significant downregulation or ↔: no significant change .

**Supplementary Figure S2. CDH11 Immunohistochemical staining pattern of invasive lobular carcinomas.** (A) CDH11 staining of human ILC as well as adjacent normal epithelium (on the far right side) and their corresponding H&E staining. Green arrows point out the whole cell staining in the pleomorphic ILC (images next to the adjacent normal epithelium) and black arrows point to a cell within the region that is magnified in the small insets. Scale bar: 45 µM. Small insets are 2.5x magnification of large images. (B) Enlarged images from the far left ILC specimen of panel A at 40x magnification (left side) and 100x magnification (right side) (C) Different field from the same representative ILC slide. Please note that epithelial cells are predominantly negative for CDH11 expression and occasionally stromal cells (lymphocytes, fibroblasts or mesenchymal cells) are CDH11 positive. Scale bar: 45 µM for left side figures and 112.5 µM for right side figures. Black arrows point to a representative cell that is shown both in 40x and 100x magnification.

**Supplementary Figure S3. Cadherin-11 knockdown localization and growth validation.**

(A) Immunofluorescence shows that CDH11 is significantly reduced in stable knockdown cell lines. Cover slips were probed with either CDH11 or beta-catenin antibody and viewed with a 20x objective on a Nikon E600 microscope. (B) CDH11 depletion significantly inhibits proliferation and (C) decreases anchorage-independent colony growth of various stable cell lines as measured using Cell Titre-Glo luminescence reagent after 5d. Columns and bars show the mean and SEM, respectively.

**Supplementary Figure S4.** **Compounds and small molecule inhibitors predicted to bind to CDH11 hydrophobic pocket show cell growth inhibition in CDH11 positive cell lines but not in control cadherin-11 negative MCF7 cells.** Dose response curves of (A) CCB, (B) DMC, (C) SD-133, (D) SD-0001A and (E) SD-0003A on CDH11 positive MDA-MB-231, BT-549 and Hs578t cells a well as their effect on control CDH11 negative MCF7 cells. Proliferation rate was measured at 96 hours with MTS assay according to the manufacturer’s protocol.

**Supplementary Figure S5.** **Workflow of small molecule inhibitor screening strategy.**
